# Parthenolide Blocks Cocaine’s Effect on Spontaneous Firing Activity of Dopaminergic Neurons in the Ventral Tegmental Area

**DOI:** 10.2174/157015911795017010

**Published:** 2011-03

**Authors:** David Schwarz, Damaris Bloom, Rocío Castro, Oné R Pagán, C.A Jiménez-Rivera

**Affiliations:** 1 Department of Biology, University of Puerto Rico, Río Piedras; 2 Department of Physiology, Universidad Central del Caribe; 3 Department of Biology West Chester University; 4 University of Puerto Rico, School of Medicine, San Juan, USA

**Keywords:** Cocaine, parthenolide, ventral tegmental, dopamine, firing activity.

## Abstract

Chronic cocaine administration leads to catecholamine reuptake inhibition which enhances reward and motivational behaviors. Ventral Tegmental Area dopaminergic (VTA DA) neuronal firing is associated with changes in reward predictive signals. Acute cocaine injections inhibit putative VTA DA cell firing in vertebrates. Parthenolide, a compound isolated from the feverfew plant (*Tanacetum parthenium)*, has been shown to substantially inhibit cocaine’s locomotion effects in a planarian animal model (Pagán *et al.,* 2008). Here we investigated the effects of parthenolide on the spontaneous firing activity of putative VTA DA neurons in anesthetized male rats (250-300g). Single-unit recordings were analyzed after intravenous (i.v.) parthenolide administration followed by 1mg/kg i.v. cocaine injection. Results showed that parthenolide at 0.125 mg/kg and 0.250mg/kg significantly blocked cocaine’s inhibitory effect on DA neuronal firing rate and bursting activity (p< 0.05, two way ANOVA). We propose that parthenolide might inhibit cocaine’s effects on VTA DA neurons *via* its interaction with a common binding site at monoamine transporters. It is suggested that parthenolide could have a potential use as an overdose antidote or therapeutic agent to cocaine intoxication.

## INTRODUCTION

The mesocorticolimbic circuit functions as the center for reward and motivation [1]. The Ventral Tegmental Area (VTA) forms part of this circuit, and is identified with modulating want and motivation [2]. VTA dopaminergic neuronal firing is associated with changes in reward predictive signals [3]. During chronic cocaine abuse, catecholamine reuptake mechanisms are inhibited, causing an increase in neurotransmitters at the synapse [4]. This increased concentration of dopamine, serotonin, or norepinephrine can have lasting effects in the form of neuroadaptations, such as enhanced reward and motivational behaviors, and serious short term effects such as cardiac arrest in susceptible individuals.

The purpose of this study was to characterize the effect of the drug known as parthenolide, a compound isolated from the feverfew plant (*Tanacetum parthenium)*, on the rat animal model. Studies have shown that parthenolide has potent anti-inflammatory effects, but recent evidence suggests a potential blockade action on cocaine’s effects in an invertebrate animal model [5, 6]. Behavioral evidence in planarians supports that parthenolide can bind to catecholamine reuptake transporters without impairing function, and this binding prevents cocaine’s effect by allosteric competition [4].

Dopamine (DA) cells in the VTA have an important role in pleasure-seeking behavior modulation [7]. They are also innervated by noradrenergic afferents and are thus modulated in part by norepinephrine [8]. Acute cocaine administration can be observed by the inhibition of VTA DA cell activity [9]. Thus, we tested parthenolide’s effect on the inhibition produced by cocaine in the rat VTA DA cells when both are injected intravenously.

## MATERIALS AND METHODS

### Animal Preparation

Sprague-Dawley male rats (250-–300 g) were used as the experimental subjects for all of these studies. The animals were housed two per cage in the colony room and maintained at a constant temperature and humidity with a 12-hr hour light/dark cycle. They were randomly assigned to the groups before the study begins. All efforts were made to minimize animal discomfort and to limit the number of animals used in this project. The procedures were approved by the Universidad Central del Caribe Institutional Animal Care and Use Committee.

Animals were anesthetized with chloral hydrate (400 mg/kg, i.p.). Afterwards the animal was placed in the stereotaxic apparatus for electrophysiological recordings. Rats were secured in a stereotaxic apparatus in the flat skull position [6]. Body temperature was maintained (37 °C) with a thermostatically controlled heating pad. A craniotomy was performed and electrodes were lowered into the VTA. Stereotaxic coordinates for the ventral tegmental area (VTA) were 3.0 to 3.4 mm anterior to lambda, 0.4 to 1.0 mm medial/lateral, and 6.0 to 8.0 mm dorsal/ventral from the cortical surface.

Drugs were administered intravenously *via* a jugular catheter. Parthenolide (Tocris) was diluted with 20% DMSO/80% dH2O until a concentrations of 0.125 mg/kg or 0.250mg/kg were achieved. Cocaine was used in a dose of 1mg/kg.

### Extracellular Recording

Single-barrel microelectrodes filled with Pontamine Sky Blue Solution 2% (PSB), tip diameter 3-6 micrometers, were used to record single unit activity in the VTA. The recording microelectrode was slowly advanced 7-8 mm until single unit activity correspondent of waveform parameters for a dopaminergic cell were observed (characteristically low (4-5 spikes/sec) firing rate, large action potential amplitude, and 1.1ms wavelength from peak to negative trough). Neuronal activity was amplified (DAM 80 amplifier, World Precision Instruments) and sent to an interface for digitalization. The interface’s output was fed to a computer for on-line waveform display (Spike 2, Cambridge Electronic Design, UK).

### Histological Verification of Recording Sites

The last recording site in an experiment was marked by iontophoresis of pontamine sky blue (2%, -7 µA for 10 minutes). The animal was euthanized, the brain removed and put in 8% formaline and stored in a refrigerator. The location of the electrodes was determined during microscopic examination of Nissl-stained, coronal brain sections, cut at 40- to 50-micron intervals in a cryostat.

### Data Analysis

Data was analyzed using Spike2 bundled software with Principal Component Analysis (PCA) for spike isolation and data mining. Microsoft Excel and GraphPad Prism 4.5 software were used for statistical analysis. Two way repeated measures ANOVA with a Bonferroni post hoc test were employed.

## RESULTS

Fig. (**1**) shows the effects of cocaine and parthenolide administration on VTA DA firing rate. Intravenous cocaine injections induced an inhibition in firing activity that peak approximately 9-12 minutes after the injection. Parthenolide, (0.125 mg/kg or 0.250 mg/kg intravenously), was administered before a single cocaine (1mg/kg,i.v.) injection. The results showed that cocaine’s inhibitory action was not affected when animals were pre-injected with saline. On the other hand, cocaine’s inhibitory action was blocked when rats were pre-injected by either dose of parthenolide, (0.125mg/kg or 0.250 mg/kg). These results were determined highly significant by a repeated measures two-way ANOVA [F(18,189) ≡ 4.05,  p < 0.0001].

When doses were compared, parthenolide at 0.125 mg/kg, seems to be more effective at inhibiting cocaine’s action, although the difference between 0.250 mg/kg and 0.125 mg/kg was not statistically significant.

Bursting analysis was conducted to investigate whether acute cocaine injections induced changes in the firing pattern of VTA DA neurons. Fig. (**2**) shows the distribution of bursting events in time as percent of the total bursting activity. The significant difference [F(18,189) ≡ 6.42, p < 0.0001] of bursting activity during the baseline period between cocaine and parthenolide treated groups (75.39% cocaine group vs 13.02% and 20.86% in parthenolide 0.125 mg/kg and 0.250 mg/kg, respectively) indicates that greater part of the cocaine group’s bursting events occurred before cocaine administration. Thus, acute intravenous cocaine administration completely inhibited bursting activity in dopaminergic VTA neurons. However, animals pre-injected with parthenolide (0.125 mg/kg or 0.250 mg/kg) maintained a relatively homogenous distribution of bursting activity. This suggests that parthenolide administration also seems to block cocaine’s inhibitory effects on bursting activity in VTA DA neurons.

The mean number of spikes per burst analysis showed that the 0.250 mg/kg parthenolide group demonstrate a decreased number of spikes after 15 minutes post injection which recovered around 30 minutes after injection [F(18,189) ≡ 2.41,  p < 0002]. The latter effect was not due to changes in the number of bursting activity as shown in Fig. (**2**). On the other hand, the 0.250 mg/kg dose induced a significant decrease in burst duration at 21 and 24 minutes after injection [F(18,189) ≡ 2.17,  p < 0.005] which might account for the decreased number of spikes observed.

## DISCUSSION

The present study demonstrates that parthenolide administration blocks the acute cocaine’s action on VTA DA neuronal firing activity. Similar to the behavioral results published by Pagán *et al.* 2008, systemic administration of parthenolide prior to a cocaine injection blocked cocaine-induced firing rate inhibition and bursting activity. Both dosages of parthenolide were effective in blocking cocaine’s action on putative VTA DA cells. This finding was consistent throughout the experiment.

The neurochemical action of cocaine is to inhibit the reuptake mechanisms of catecholamines causing an increase in neurotransmitters at the synapse [4]. It has been shown that acute administration of psychostimulant drugs such as cocaine decrease VTA DA neuronal activity. This action is likely due to the fact that psychostimulants drugs enhance extracellular concentrations of dopamine at the somatodendritic area resulting in an activation of impulse-regulating autoreceptors thus inhibiting neuronal firing [10, 11]. Changes in VTA DA cell activity while the drug of abuse is onboard are important for triggering the enhancement of neuronal activity observed after withdrawal from drug exposure. Eventually this increase in VTA neuronal firing is critical in the development of cocaine sensitization [12].

Parthenolide has been shown to play a role in other mechanisms, such as inhibiting the NF-κB cell signaling pathway [13], suggesting a drug’s interaction with other mechanisms that might not be in operation at lower dosages. It is likely that parthenolide at a dose of 0.125 mg/kg is more effective at exerting its effect on cocaine’s mechanism of action. Interestingly, neurons treated with parthenolide at 0.250 mg/kg had a reduction in burst duration, which correlated with a diminution in the number of spikes per burst. Further studies including dose response curves could serve to clear this issue.

Previous experiments [4] showed that cocaine and parthenolide do not interact directly; instead, their effect seems to be localized to a common binding site on the planarian nervous system. Based on this previous study and on the work presented here, we propose that parthenolide actively competes with cocaine, displacing the drug from inhibiting the transporter and allowing it to function normally. These observed behavioral and neurophysiological interactions of parthenolide and cocaine are consistent with the idea of parthenolide acting as a dopamine-sparing, cocaine antagonist (DSCA) [14]. DSCA’s are hypothesized to bind to the dopamine-cocaine sites on the dopamine transporter in such a way that block cocaine’s interaction with the transporter, while leaving dopamine interactions with the transporter unaffectaed [10]. Binding studies, could further clarify parthenolide’s mechanism of action. A positive result could further clarify parthenolide’s role in the inhibition of cocaine’s effect.

Since high concentrations of cocaine acutely can be harmful or even lethal to an organism, a compound that acts at a common binding site displacing cocaine from it could have utility as an overdose antidote or therapeutic agent.

## Figures and Tables

**Fig. (1).**
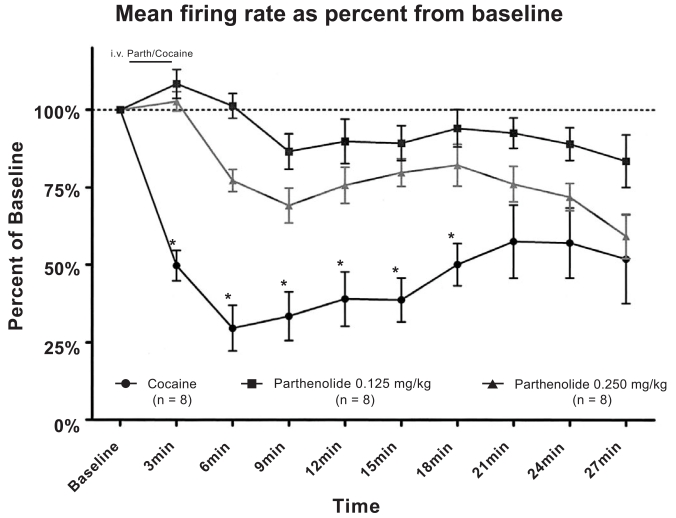
Effects of parthenolide on VTADA neuronal firing rate. Parthenolide administration (0.125 mg/kg, i.v. or 0.250 mg/kg, i.v.) blocked cocaine’s (1 mg/kg, i.v.) inhibitory actions on VTADA firing activity. (Two way ANOVA, P<0.0001).

**Fig. (2).**
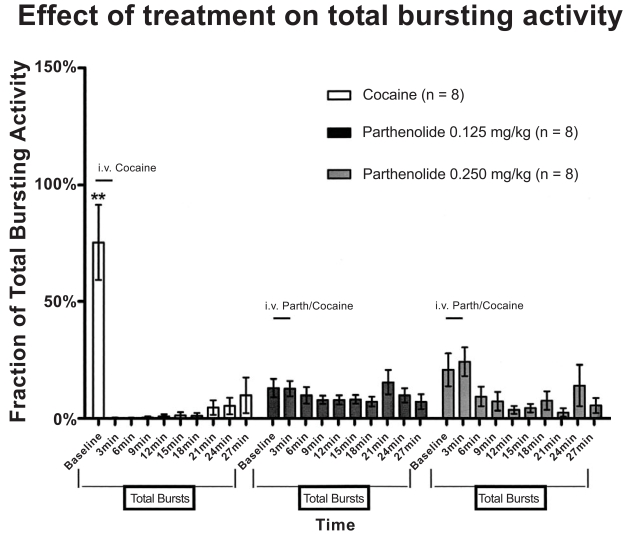
Distribution of bursting events in time as percent of the total bursting activity. Cocaine administration almost totally inhibited bursting activity of VTADA cells. Most part of cocaine’s bursting events occurred before cocaine administration. Animals previously injected with parthenolide maintained a relatively homogeneous distribution of bursting activity. There was a significant difference in bursting activity between cocaine and parthenolide treated groups (Two way ANOVA, P<0.0001).
